# The accuracy of radiology speech recognition reports in a multilingual South African teaching hospital

**DOI:** 10.1186/s12880-015-0048-1

**Published:** 2015-03-04

**Authors:** Jacqueline du Toit, Retha Hattingh, Richard Pitcher

**Affiliations:** Department of Diagnostic Radiology, Tygerberg Academic Hospital, Stellenbosch University, Francie van Zyl Avenue, Cape Town, 7700 South Africa

**Keywords:** Speech recognition, Transcriptionist, Error rate, Radiology reporting

## Abstract

**Background:**

Speech recognition (SR) technology, the process whereby spoken words are converted to digital text, has been used in radiology reporting since 1981. It was initially anticipated that SR would dominate radiology reporting, with claims of up to 99% accuracy, reduced turnaround times and significant cost savings. However, expectations have not yet been realised. The limited data available suggest SR reports have significantly higher levels of inaccuracy than traditional dictation transcription (DT) reports, as well as incurring greater aggregate costs.

There has been little work on the clinical significance of such errors, however, and little is known of the impact of reporter seniority on the generation of errors, or the influence of system familiarity on reducing error rates.

Furthermore, there have been conflicting findings on the accuracy of SR amongst users with English as first- and second-language respectively.

**Methods:**

The aim of the study was to compare the accuracy of SR and DT reports in a resource-limited setting. The first 300 SR and the first 300 DT reports generated during March 2010 were retrieved from the hospital’s PACS, and reviewed by a single observer. Text errors were identified, and then classified as either clinically significant or insignificant based on their potential impact on patient management. In addition, a follow-up analysis was conducted exactly 4 years later.

**Results:**

Of the original 300 SR reports analysed, 25.6% contained errors, with 9.6% being clinically significant. Only 9.3% of the DT reports contained errors, 2.3% having potential clinical impact. Both the overall difference in SR and DT error rates, and the difference in ‘clinically significant’ error rates (9.6% vs. 2.3%) were statistically significant. In the follow-up study, the overall SR error rate was strikingly similar at 24.3%, 6% being clinically significant.

Radiologists with second-language English were more likely to generate reports containing errors, but level of seniority had no bearing.

**Conclusion:**

SR technology consistently increased inaccuracies in Tygerberg Hospital (TBH) radiology reports, thereby potentially compromising patient care. Awareness of increased error rates in SR reports, particularly amongst those transcribing in a second-language, is important for effective implementation of SR in a multilingual healthcare environment.

## Background

Effective communication plays a pivotal role in modern radiological practice, with the generation of accurate reports being integral to optimal patient care [1].

The radiology report has been shown to be the most important determinant of a radiologist’s stature amongst clinical colleagues [2].

Speech recognition (SR) technology, the process whereby the spoken word is converted to digital text, has been used in radiology reporting since 1981 [3]. The earliest systems did not enhance efficiency, requiring users to pause between individual words. However, with on-going software development, the first continuous speech programmes evolved in 1994 and by 1999, state of the art systems were claiming up to 99% accuracy [4], reduced report turnaround times [5], and significant cost savings [6-9]. It appeared that SR was destined to dominate radiology reporting.

Despite initial promise, the limited available data suggest that SR reports contain more errors than those generated by traditional dictation transcription (DT) [7-10]. SR reports have been shown to require thorough proofreading and editing [7,8], resulting in user frustration [6] and dissatisfaction and an increase in overall reporting costs when radiologists’ editing time is incorporated [7].

It has also been shown that radiologists consistently tend to underestimate their own error rates [11]. In a questionnaire conducted by Quint [11], the majority of radiologists within a department estimated their individual report error rates to be less than 10%, whereas the overall error rate was found to be 22%.

Typical SR errors are wrong-word substitution, nonsense phrases, and missing words [11]. Examples include ‘the right sided chest and has been removed’, ‘the renal pancreas appears normal’ [9] and ‘ptosis of the right ventricular physiology’ [11]. There has, however, been little work on the clinical significance of such errors [9].

In addition, there have been no previous studies of the impact of system familiarity on error rates.

There are also no data on the utilisation of SR in low and middle-income countries (LMICs), where a scarcity of experienced medical transcriptionists in the face of burgeoning demands for radiological services and the increasing complexity of investigations make SR an attractive option, and potentially the only solution, for increasing transcription capacity.

Furthermore, there have been conflicting findings on the accuracy of SR amongst users with English as first language and second language respectively. While North American studies by Basma [10] and Quint [11] found that home language did not impact the accuracy of English SR reporting, McGurk [9] found that users with English as a second language generated significantly more SR errors.

The accuracy of SR reports generated in a language other than the user’s mother-tongue is an important consideration in modern radiological practice, where digital imaging has contributed to globalisation of radiological services. It is also an important consideration in multilingual societies such as South Africa.

Our institution. Tygerberg Hospital (TBH), is a 1386-bed tertiary-level public-sector teaching hospital in Cape Town, South Africa, affiliated to the Faculty of Medicine and Health Sciences of Stellenbosch University. At the time of this study, the radiology department was performing in excess of 160,000 examinations annually, and was staffed by 10 consultant radiologists and 24 registrars from diverse backgrounds. Five different home languages, including English, were represented amongst the radiology consultant and registrar complement. The department had just two medical transcriptionists, both with many years of experience, although neither had English as first language.

The Philips Speech Magic (version 6.1) English language SR system, with a radiology-specific vocabulary, was introduced into the TBH radiology department in January 2010, as part of the phased roll-out of a *Philips* PACS-RIS (Picture archiving and communication system – Radiology information system) solution.

The implementation of SR was preceded by a structured and comprehensive radiologist training programme, overseen by a *Philips* application specialist. Only once training had been completed, were users afforded access to their customised SR profiles. Training included detailed instruction on proofreading and self-editing of dictated reports.

Training was conducted in the knowledge that state-of-the-art SR systems have the capacity to configure a customised user voice-profile in less than 10 minutes [4]. SR reports were produced by a handheld Philips speech microphone and signed out after editing. There was no double reading. Dictated reports were generated using a standard dictaphone and transcribed by the departmental transcriptionists. Reporting radiologists checked reports for possible errors prior to sign off. Users had the option of using either SR or DT.

### Aim

The aim of this study was to compare the accuracy of English SR and DT reports in a multilingual radiology department, set in a resource-limited healthcare environment. Reports were analysed at the time of SR introduction, and again four years later, to assess the impact of SR familiarity on error rates. The clinical significance of errors, the impact of mother-tongue and reporter seniority were also assessed.

### Methodology

The study was conducted after SR had been in full clinical use for 6 weeks. The first 300 SR and the first 300 DT reports generated at TBH during March 2010 were retrieved from the hospital’s PACS, and reviewed by a single observer. Each report was scrutinised for the presence of text errors which were recorded on a customised data sheet.

A second set of SR reports were then retrieved exactly 4 years after the initial analysis and assessed according to the same methodology. During the intervening four years, a single hardware upgrade was made and a new, larger, SR server installed. No software changes were made, and existing licences and user login credentials were migrated to the new server. DT was no longer in routine use at the time of the follow-up study.

All documented errors were then classified as ‘clinically significant’ or ‘insignificant’ by consensus of three senior radiologists. A clinically significant error was defined as any error with the potential to be unclear to clinicians, or to impact patient management. Examples from our study include ‘anterior and posterior in the osseous artery’ (nonsense phrase) and ‘Calcified haematoma in the right frontal lobe consistent with.’ (word omission).

The reporter’s first language and level of seniority were also recorded.

Ethics approval was obtained from the Health Research Ethics Committee of Stellenbosch University, and patient confidentiality protected through the use of unique study numbers.

MS Excel was used to capture the data and STATISTICA version 9 (www.statsoft.com) used to analyse the data.

## Results

The study included plain film, ultrasound, fluoroscopy, mammography, computed tomography and magnetic resonance imaging reports.

Seventy-seven (25.6%) of the 300 SR reports were found to contain errors, with 29 of these (9.6%) deemed clinically significant. Only 28 (9.3%) of the 300 DT reports contained errors, with 7 (2.3%) having potential clinical impact.

Both the overall difference in SR and DT error rates, and the difference in ‘clinically significant’ error rates (9.6% vs. 2.3%), achieved statistical significance (p = 000000 and p = 0.00016 respectively).

In the follow-up analysis, 73 (24.3%) of the 300 SR reports contained errors – a striking similar result to the original error rate of 25.6%. The proportion of clinically significant errors was slightly smaller at 6% - not a statistically significant difference.

The overall SR error rate for radiologists with English as home language was 19.5%, compared to 27.6% for radiologists with English as second language.

Junior registrars (those in their first 2 years of radiology training) were responsible for the generation of 44.5% the SR reports, but level of seniority had no significant bearing on the resultant error rates.

In cases of wrong-word substitution, the intended meaning could potentially be distorted, resulting in inappropriate patient management. For example, pathology within ‘bowel’ would be investigated in a different manner to that related to ‘bile’. (See Table 1 for further examples) Similarly, instances of word omission such as ‘intracranial haemorrhage’ rather than ‘no intracranial haemorrhage’ may conceivably have serious consequences.

**Table 1:** **Examples of VR ‘wrong-word substitution’**

**Error**	**Intended**
Speculation	Spiculation
Brick areas	A Bricker’s
Into plate sclerosis	End-plate sclerosis
Raising cisterns	Basal cisterns
Femoral focus	Femoral artery
Impression is made	Comparison is made
Severe carcinoma	Liver carcinoma
See 5/6	C 5/6
Airspace dislocation	Airspace opacification
No boy metastases	No bony metastases
ETT in situ, appears no	ETT in situ, appears low
Lesions are collections	Lesions or collections
Cardiothymic	Caudothalamic
Dense fracture	Dens fracture
Transaction	Transection

## Discussion

There are limited data on the utilisation of SR technology in radiology [12]. Previous studies have been conducted in well-resourced healthcare environments. Our report of SR in a multilingual department in a resource-limited setting therefore contributes important new insights into the broad challenges of SR implementation. Our study was underpinned by the knowledge that the appointment of additional staff, including transcriptionists, is severely curtailed in resource-limited environments. Furthermore, well-trained and experienced transcriptionists are not easily recruited into the public sector, given the more lucrative opportunities in private healthcare. SR may thus represent the only option to enhance transcription capacity in resource-limited environments, where there are great pressures to meet the burgeoning demands of radiological service outputs. In the resource-limited setting, the debate is thus not ***if***, but rather ***how,*** SR technology can be successfully and safely implemented.

There is a trend to globalisation of radiological services [13,14]. There is also a drive to increased diversity amongst medical staff and students in training institutions internationally [15-17]. Multilingual radiology departments are thus encountered with increasing frequency. Knowledge of the implications of second-language SR usage is thus important.

The overall SR error rate of 25% recorded in our multilingual department at TBH (within 6 weeks of the introduction of the technology) is comparable to that documented by Quint (22%) and Basma (23%) in well-resourced environments where the technology was in established use. It is considerably better than that recorded by Pezzullo (35%), but falls well short of the accuracy achieved by McGurk and co-workers (4.8%). Nonetheless, it is clear that the overall TBH SR error rate falls within the range of international norms.

It is interesting that the error rate in a follow-up SR analysis corresponded almost exactly with the original study, calling into question the notion that SR accuracy improves with ongoing usage.

Notwithstanding this, the finding that up to 10% of SR reports contain clinically significant errors, and that users with English as second language may have higher error rates, are important findings that will have to be addressed effectively if SR is to assume a meaningful role in the modern radiology department. In this study we have thus confirmed previous findings that SR potentially compromises the effective communication that is central to the role of the radiologist and thus may contribute to compromised patient care. This has important medico-legal implications.

It has been suggested that 95% accuracy be the standard of care for specialist radiological reporting in the emergency setting [18]. We recommend that similar standards apply for transcription services, whether SR or DT. We suggest that departments introduce interventions aimed at improving the accuracy of transcription services and that quality assurance measures be implemented to monitor error rates.

Whether major or inconsequential, report errors are evidence of cursory editing. Carelessness, time pressure and the ‘recency’ phenomenon, where errors are less likely to be detected on immediate review, are all thought to be possible causative factors. [19] ^‘^Ill-conceived incentives’ (e.g. rewarding speed over accuracy) may also be responsible [5]. Furthermore, in contrast to human transcriptionists, the speech recognition software currently available is not “context-sensitive”, and therefore lacks the ability to make judgements [20].

In order to improve report accuracy, radiologists would need to be more thorough in their report editing, thereby defeating the cost benefit objective, and further reducing productivity [12].

It has been suggested that the use of intensive individual feedback within a peer group may provide insight into patterns of SR errors unique to a particular radiologist. The implementation of peer review may also provide motivation for more careful proofreading of reports, and thereby reduce final error rates [19].

A follow-up study where ‘self-edited’ reports are compared to those undergoing peer-review prior to final sign-off is suggested. In addition, error rates could be re-analysed once the results of this study were made available to the relevant group of radiologists.

‘Back-end’ editing of SR reports performed by medical transcriptionists, rather than radiologists, is yet another potential solution which may prove considerably more cost-effective. It is possible that even the most sophisticated SR systems will never be accurate enough to completely eliminate the need for human review.

### Study limitations

Although the study sample numbers are small, they remain comparable to other studies investigating the accuracy of SR.

In view of the relatively small numbers, we did not conduct sub-analyses of error rates in specific modalities. We thus assumed similar error rates across the modalities and further work will be required to assess whether there are modalities at particular risk for high SR error rates.

The “clinically significant” errors may have been over-or under-reported; there are no clear guidelines as to what constitutes a clinically significant error. The consensus decision of our study radiologists could perhaps have been different had different radiologists were involved.

## Conclusion

We have confirmed previous findings that the use of SR consistently results in significantly higher error rates than DT and thereby, may compromise patient care.

We have shown that, in our setting, SR users transcribing in a second language are at increased risk of generating errors.

We have suggested acceptable transcription error rates and discussed possible interventions for improving SR accuracy.
